# Hypergraph-Driven Heterogeneous Spatial Relationship Learning for Remote Sensing Segmentation

**DOI:** 10.3390/jimaging12090404

**Published:** 2026-08-27

**Authors:** Qihao Zhang, Lankun Peng, Feiyang Hu, Xiaoming Xi

**Affiliations:** School of Computer and Artificial Intelligence, Shandong Jianzhu University, Jinan 250101, China; qihaozhang.mail@gmail.com (Q.Z.); penglankun24@gmail.com (L.P.); hufeiyang0616@163.com (F.H.)

**Keywords:** heterogeneous spatial relationships, higher-order relationships, hypergraph learning, remote sensing, semantic segmentation

## Abstract

Remote sensing semantic segmentation is critical for extracting fine-grained spatial information in applications such as urban planning and environmental monitoring. However, existing methods face significant challenges in modeling heterogeneous spatial relationships within complex urban scenes, where semantically related regions are spatially dispersed yet functionally interdependent. Conventional convolutional neural networks exhibit limited receptive fields that fail to capture long-range dependencies, while Transformer-based approaches capture global dependencies but do not explicitly model regional heterogeneity, leading to blurred boundaries and category confusion. To address these limitations, this paper proposes a novel multi-relational-aware segmentation framework that leverages hypergraph theory to dynamically model higher-order semantic groupings across non-adjacent regions. The core innovation lies in a hypergraph structure learning unit that employs fuzzy clustering to partition multi-scale features into adaptive hyperedge sets, enabling joint representation of topological associations and functional dependencies among spatially distributed entities. Additionally, a multi-scale co-modeling strategy integrates stochastic feature masking with weighted fusion to bridge semantic abstraction and spatial localization. Experiments demonstrate that the proposed method achieves state-of-the-art mIoU performance on the LoveDA, Vaihingen, and Potsdam datasets, obtaining mIoU scores of 54.70%, 85.01%, and 87.64%, with improvements of 0.30%, 0.91%, and 0.08%, respectively, over the best existing methods.

## 1. Introduction

Remote sensing semantic segmentation [1,2] plays a pivotal role in extracting fine-grained spatial information from high-resolution satellite or aerial imagery, serving as a cornerstone for applications such as urban planning, environmental monitoring, disaster management, and land use analysis. The ability to accurately distinguish and classify various land cover types—such as buildings, roads, vegetation, and water bodies—enables dynamic tracking of the evolution of urban systems and environmental changes [3]. With advancements in remote sensing technology, the resolution of high-resolution imagery has reached sub-meter levels (e.g., 0.3 m for WorldView-3). However, fine-grained categories [4] in complex urban scenes (e.g., distinctions between old and new building types, industrial and storage zones) and spatial heterogeneity (e.g., interwoven distributions of dense urban areas and green spaces) continue to pose significant challenges to existing methods. Traditional approaches often encounter performance bottlenecks in such scenarios due to ambiguities in local features (e.g., texture similarities between shadows and buildings) or the absence of long-range semantic dependencies (e.g., functional relationships between roads and distant buildings) [5].

**Figure 1:**
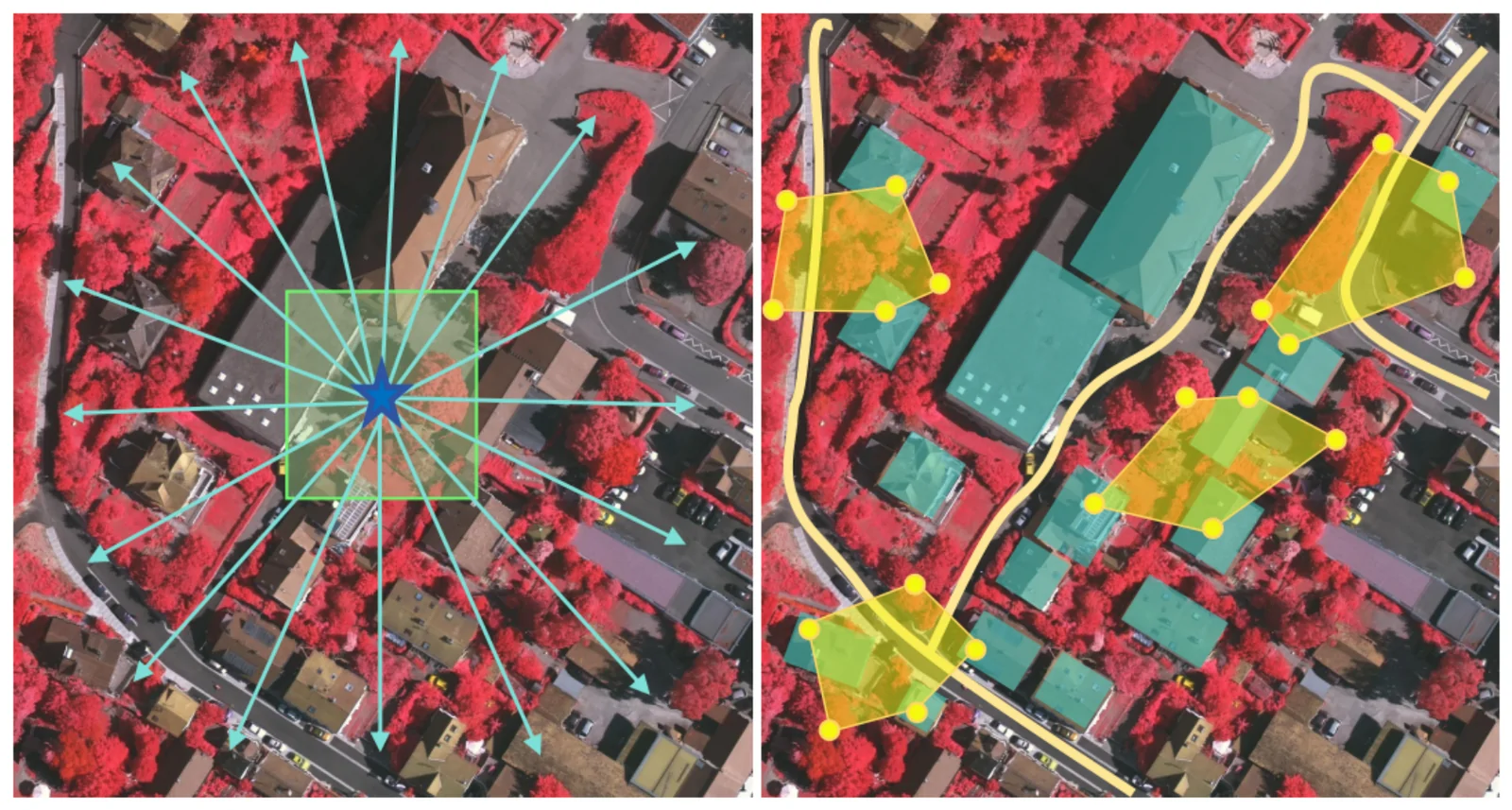
Limitations of conventional architectures in modeling heterogeneous spatial relationships for remote sensing segmentation. (**Left**): CNNs (fixed-range receptive field, green box) and Transformers (global self-attention, cyan arrows) fail to capture semantic dependencies between spatially dispersed regions (e.g., distant buildings and roads). The blue star denotes the reference region used to illustrate its interactions with other spatial regions. (**Right**): Higher-order functional semantic grouping through hyperedges (yellow edges) requires joint modeling of multiple non-adjacent spatial regions (marked by yellow nodes) to achieve accurate segmentation, which cannot be addressed by conventional methods.

Among these challenges, heterogeneous relationships between spatially dispersed but semantically related regions represent a fundamental yet underexplored issue in remote sensing segmentation. In complex urban environments, different spatial regions often exhibit intricate semantic dependencies that extend beyond their immediate proximity. For instance, the functional type of a building (e.g., residential or commercial) can be inferred from its spatial adjacency to roads of specific widths or vegetation patterns, while water bodies and urban structures often form contextual clusters that define land use categories. As illustrated in Figure 1, conventional architectures are inherently constrained in modeling heterogeneous spatial relationships. The localized receptive fields of traditional convolutional neural networks [6] (CNNs), constrained by fixed ranges, struggle to model semantic dependencies between distant regions (e.g., roads and remote buildings). Although Transformers [7] can capture global correlations through self-attention mechanisms, standard self-attention does not explicitly model regional heterogeneity, such as semantic disparities or spatial layout variations among regions. This limitation may lead to suboptimal performance in preserving object boundaries and distinguishing visually similar categories.

Given these limitations, this study aims to address the dual challenges of preserving local details and understanding global context in remote sensing segmentation. While existing hybrid architectures [8] (e.g., UNetFormer) have significantly enhanced the representation capacity of complex scenes by integrating CNN-based local feature extraction with Transformer-based global attention mechanisms, their feature aggregation frameworks still exhibit limitations in explicitly modeling heterogeneous relationships among spatially distributed regions. Although these architectures can adaptively aggregate contextual information, they generally do not construct explicit region-level relational structures for organizing interactions among multiple semantically related regions.

Specifically, hybrid models typically capture local spatial patterns through convolutional operations and global contextual correlations through self-attention. However, such feature-level aggregation does not explicitly organize multiple spatially separated regions into structured relational groups. For example, semantic associations between roads and distant buildings may not be sufficiently represented by local convolutional features, while global self-attention may capture broad correlations without explicitly distinguishing region-specific heterogeneity, such as differences in semantic roles or spatial layouts. Consequently, heterogeneous relationships among spatially distributed regions may remain insufficiently represented, which can contribute to boundary ambiguity and category confusion. Therefore, explicitly constructing adaptive region-level relational structures for modeling higher-order heterogeneous interactions remains an important challenge in remote sensing semantic segmentation.

To overcome the limitations of static modeling frameworks, this study proposes the introduction of hypergraphs [9] as a dynamic modeling tool for heterogeneous spatial relationships. The core motivation lies in the fact that traditional graph structures or attention mechanisms can only model binary node relationships (e.g., interactions between pixel pairs or region pairs), whereas complex semantic associations in remote sensing scenes often involve higher-order relationships among multiple nodes. For instance, the functional category of a building is jointly defined by its roof structure, surrounding road networks, and the distribution patterns of adjacent vegetation—a multi-element collaborative semantic representation that cannot be fully captured through simple binary relationship modeling. Hypergraphs, through the mathematical structure of hyperedges, naturally enable the joint modeling of multiple spatial regions or feature nodes as a unified entity, thereby explicitly encoding synergistic constraints between distant regions (e.g., topological associations between roads and buildings) or semantic dependencies of local structures (e.g., geometric relationships between building contours and shadows). Compared to graph convolutional networks [10] (GCNs) or self-attention mechanisms, hypergraphs excel in modeling higher-order relationships—bypassing the need to approximate complex interactions through combinations of binary edges and instead directly defining joint probability distributions or feature aggregation rules among multiple nodes via hyperedges. This characteristic allows hypergraphs to more accurately characterize spatially dispersed but semantically coupled regional clusters (e.g., the coordinated layout of airport runways and aprons) or distinguish visually similar yet functionally distinct categories (e.g., differentiating between industrial and storage building clusters). Thus, the incorporation of hypergraphs addresses a representational limitation of conventional pairwise relational modeling by providing an adaptive mechanism for higher-order feature aggregation.

To achieve these objectives, this study proposes a multi-relational-aware semantic segmentation framework, the Hypergraph Network (HyperNet) for remote sensing segmentation, designed to address the modeling of heterogeneous spatial relationships in remote sensing scenes. Its core architecture is based on a hybrid Transformer-CNN encoder-decoder structure, which extracts multi-scale features through the synergistic design of global context modeling and local detail capture [11]. To transcend the limitations of static feature aggregation in conventional methods, we introduce a hypergraph modeling module dedicated to capturing heterogeneous spatial relationships, with its core innovation lying in the design of a hypergraph structure learning unit. This module employs a fuzzy clustering algorithm to partition multi-scale feature nodes (e.g., spatial regions or semantic segments) extracted by the decoder into dynamic hyperedge sets, where each hyperedge can jointly model higher-order relationships among multiple nodes (e.g., topological associations between building contours and road networks, semantic dependencies between vegetation distribution and functional zones). Through hypergraph convolution mechanisms, node features undergo non-local information propagation under the constraints of hyperedges, thereby explicitly encoding multi-faceted relationships among geographical entities (e.g., adjacency, exclusion, or functional synergy). Additionally, we propose a multi-scale co-modeling strategy: dynamic masking of encoder features via a stochastic perturbation mechanism forces the model to learn more robust cross-scale representations, while a weighted feature fusion module adaptively integrates perturbed deep features with shallow decoder details to bridge the representational gap between semantic abstraction and spatial localization. By leveraging the synergy between higher-order relationship modeling via hypergraphs and dynamic multi-scale feature interactions, our model provides novel methodological support for the precise parsing of complex spatial semantics in remote sensing scenes.

Our contributions are threefold:

- We propose a hypergraph-driven remote sensing segmentation framework, termed HyperNet, which jointly models heterogeneous spatial relationships and multi-scale feature representations for accurate segmentation of complex remote sensing scenes.
- We develop a Hypergraph-Driven Functional Semantics Module (HFSM) and a Multi-Scale Co-Modeling Module (MCM). HFSM dynamically constructs hyperedges through fuzzy clustering to capture higher-order semantic relationships, while MCM combines stochastic feature masking with adaptive weighted fusion to enhance cross-scale feature robustness.
- Extensive mechanism-level and experimental analyses demonstrate the effectiveness of the proposed HyperNet. Specifically, HyperNet achieves state-of-the-art mIoU scores of 54.70%, 85.01%, and 87.64% on LoveDA, Vaihingen, and Potsdam, outperforming existing methods by 0.30%, 0.91%, and 0.08%, respectively.

## 2. Related Work

Existing remote sensing segmentation methods mainly rely on CNN-based and Transformer-based architectures. CNN-based methods focus on local feature extraction and multi-scale representation, while Transformer-based methods enhance global context modeling. However, explicit modeling of heterogeneous spatial relationships among multiple regions remains limited.

### 2.1. CNN-Based Methods

The fully convolutional network (FCN) [12] revolutionized semantic segmentation by enabling end-to-end pixel-wise classification, rapidly becoming the cornerstone of remote sensing applications. However, its simplified decoder architecture inherently struggles to preserve spatial details, leading to coarse segmentation results in complex remote scenes. This limitation spurred the development of more sophisticated encoder-decoder frameworks.

The U-Net [13] architecture addressed this challenge by introducing symmetric skip connections to integrate low-level spatial details with high-level semantic features. This design significantly improved segmentation accuracy in medical imaging and was quickly adopted in remote sensing. For example, U-Net achieved over 85% accuracy in building and road extraction on the ISPRS Vaihingen dataset. Recent advancements have further optimized U-Net’s skip connections and decoders. RCFSNet [14] addresses these limitations. Another innovation, CM-UNet [15], replaces traditional skip connections with a multi-scale attention aggregation module (MSAA). This enhancement improves feature fusion across resolutions and achieves state-of-the-art performance on high-resolution satellite imagery.

Despite these improvements, traditional CNNs remain limited by their inability to model global dependencies. In urban scenes with dense buildings and roads, CNNs with restricted receptive fields struggle to distinguish complex patterns. To overcome this, multi-scale feature aggregation and attention mechanisms have been widely explored. By integrating attention gates, Attention U-Net [16] focuses on salient spatial features to enhance segmentation accuracy in difficult areas. DANet [17] utilizes dual attention modules for spatial and channel-wise feature refinement, achieving superior performance on various segmentation benchmarks. Lightweight architectures such as BiSeNetv2 [18] balance accuracy and speed by combining spatial detail with context information through a two-branch structure, suitable for real-time remote sensing applications. MST-DeepLabv3+ [19] introduces the SENet attention mechanism module and transfer learning to better enhance semantic segmentation accuracy. LOGCAN++ [20] introduces GCA attention modules to capture global representations for class-level context modeling, thus reducing background noise interference and improving segmentation mIoU by 1.23% in the Vaihingen dataset. These methods improve local representation through multi-scale aggregation and attention mechanisms. However, CNN-based architectures still rely mainly on local feature extraction and feature-level enhancement. Their ability to model semantic relationships between spatially separated regions remains limited.

### 2.2. Transformer-Based Methods

Unlike conventional Convolutional Neural Network architectures, Transformer-based methods reformulate two-dimensional image processing tasks as the one-dimensional sequence modeling problems [21]. Leveraging its robust sequence-to-sequence learning capabilities, the Transformer architecture exhibits superior performance in capturing global contextual relationships compared to attention mechanisms operating in isolation [22]. This enhanced modeling capacity has enabled Transformer-based approaches to achieve state-of-the-art performance across core computer vision applications, including but not limited to image classification [23], object detection [24], and semantic segmentation [25].

Transformer-based methods for remote sensing segmentation can be broadly categorized into two groups. The first category consists of pure Transformer architectures, exemplified by ResU-Former [26], which integrates Swin residual Transformer blocks for multi-scale feature fusion. This approach balances visual-semantic spatial information via hierarchical structures and employs dynamic sparse training to reduce complexity. A notable limitation of this approach is its lack of CNN’s local feature extraction capabilities, often leading to deteriorated segmentation accuracy for small targets [27]. The second category comprises hybrid CNN-Transformer architectures that address the limitations of pure Transformer designs [28]. For instance, SSNet innovatively integrates Feature Fusion and Feature Injection Modules (FFM & FIM), achieving mIoU scores of 61.93% on the WHDLD and 77.67% on the Potsdam datasets by constructing a dual-branch cooperative mechanism, which effectively enhances the discrimination of similar objects and small targets. UNetFormer [8] employs CNN-based feature encoding and Transformer-based decoding. However, these approaches mainly focus on global context modeling and feature fusion. They do not explicitly model heterogeneous spatial relationships among multiple regions. This limitation reduces their ability to represent complex spatial dependencies in remote sensing scenes.

### 2.3. Graph-Based and Hypergraph-Based Methods

Graph-based methods have been explored to model structural relationships in remote sensing images. KGGCN [29] constructed graphs based on geographic objects and their topological relationships for semantic segmentation. A hierarchical class graph framework [30] was introduced to capture structured correlations among different ground-object categories. More recently, MGF-GCN [31] employed graph convolution to model spatial correlations between multimodal remote sensing features.

Although graph-based methods improve relational representation, conventional graphs mainly model relationships through pairwise edges between nodes. Hypergraphs extend this representation by associating multiple nodes within a hyperedge. DNHGNN [32] combines multi-scale superpixel segmentation with hypergraph convolution to capture higher-order relationships among remote sensing objects for change detection. HGBT [33] further introduces hypergraph modeling into a Transformer architecture to capture high-order correlations and local topology for remote sensing semantic segmentation.

These methods demonstrate the potential of graph and hypergraph learning for remote sensing interpretation. However, adaptive modeling of heterogeneous spatial relationships among distributed regions remains insufficiently explored. In contrast, HyperNet constructs adaptive hyperedges from feature representations through fuzzy clustering. This enables higher-order relationships among multiple spatial regions to be dynamically modeled.

Overall, existing methods have improved local and global feature representation, while graph and hypergraph learning further enhances relational modeling. However, adaptive modeling of heterogeneous spatial relationships among distributed regions remains insufficiently explored. The robustness of cross-scale feature interactions also requires further investigation. These limitations motivate the design of HyperNet. HFSM models heterogeneous spatial relationships, while MCM enhances robust multi-scale feature interaction.

## 3. Methodology

### 3.1. Mechanism Analysis of Hypergraph Modeling

Before introducing the specific methodological design, we analyze the mechanism of hypergraph modeling from the perspective of feature aggregation and relational propagation. Unlike conventional pairwise relations, a hyperedge can associate multiple feature nodes within a unified structure. This property enables information exchange among spatially distributed but semantically related regions. Therefore, hypergraph modeling provides a natural way to represent higher-order relationships in complex remote sensing scenes.

#### 3.1.1. Parameterized Feature Aggregation for Hypergraphs

Let the feature aggregation operation of the hypergraph model be defined as

(1) $$Z_{v}=\sigma \left(H\mathrm{softmax}\left(H^{T}XW_{e}\right)W_{v}\right).$$

where $H\in R^{N\times M}$ is the incidence matrix, $W_{e}$ and $W_{v}$ are learnable parameters, and $X\in R^{N\times D}$ is the node feature matrix. Each column of H describes the associations between feature nodes and a hyperedge.

The aggregation consists of two stages. First, $H^{T}XW_{e}$ aggregates the projected node features into hyperedge representations. The hyperedge features are then propagated back to their associated nodes through $HZ_{e}W_{v}$. This node-to-hyperedge and hyperedge-to-node process enables related nodes to exchange information through shared hyperedges.

Unlike conventional pairwise interactions, one hyperedge can connect multiple nodes simultaneously. Thus, the resulting feature representation contains group-level contextual information from several related regions. This property provides the basic mechanism for higher-order relationship modeling in HyperNet.

#### 3.1.2. Intuitive Effect of Shared Hyperedge Aggregation

Nodes belonging to the same hyperedge participate in a shared feature aggregation process. Their representations are jointly integrated into a common hyperedge feature and then redistributed to the associated nodes. This process encourages semantically related nodes to exchange complementary information and learn more consistent representations.

From an intuitive perspective, shared aggregation can also reduce the influence of isolated local feature variations. A node is no longer updated only according to its individual representation. Instead, its updated feature also incorporates information from other related nodes in the same hyperedge. This group-wise interaction may lead to smoother feature updates during training.

However, this effect depends on the learned hypergraph structure, feature distribution, and optimization process. Therefore, we interpret it as a mechanism-level explanation rather than a general guarantee of gradient variance reduction.

#### 3.1.3. Optimization Interpretation

The higher-order connections introduced by hypergraphs provide direct information propagation among multiple related nodes. This property is particularly useful for remote sensing images, where semantically related regions may be spatially separated. Through shared hyperedges, information can be exchanged across these regions without relying solely on isolated local interactions.

Such relational propagation can reduce information fragmentation among related regions. It can also provide more consistent contextual information for feature learning. From an optimization perspective, these properties may facilitate a more stable learning process and improve feature interaction during training.

Nevertheless, the convergence behavior of a deep segmentation network is affected by many factors, including the network architecture, loss function, optimizer, and learned hypergraph structure. Therefore, we do not assume that hypergraph modeling guarantees a faster convergence rate. Its practical optimization behavior is evaluated through the experimental results.

#### 3.1.4. Higher-Order Connectivity Interpretation

Conventional graphs generally describe relationships through pairwise edges between nodes. In contrast, a hyperedge can directly connect multiple nodes. This higher-order connectivity is suitable for representing complex dependencies among geographical entities in remote sensing scenes.

For example, the semantic interpretation of a building may depend jointly on its surrounding road network and vegetation distribution. These relationships can be represented by a hyperedge as

(2) $$e_{m}=\left\{v_{\mathrm{building}},v_{\mathrm{road}},v_{\mathrm{vegetation}}\right\}.$$

The associated nodes can first contribute information to the shared hyperedge and then receive the aggregated contextual information. In this way, the relationships among multiple regions are modeled within a unified structure rather than through several independent pairwise connections.

The effectiveness of this propagation depends on the quality of the constructed hyperedges and their association strengths. Therefore, we do not assume that hypergraphs universally possess superior spectral properties to conventional graphs. Instead, their advantage in this work lies in the explicit representation and propagation of higher-order information among multiple spatial regions.

In summary, the above analysis provides an intuitive interpretation of hypergraph modeling rather than formal optimization guarantees. Hyperedges organize multiple related spatial regions into shared relational structures and enable group-wise information propagation. These properties facilitate the modeling of higher-order heterogeneous spatial relationships in remote sensing scenes. Based on this motivation, we further design the Hypergraph-Driven Functional Semantics Module in Section 3.5 to dynamically construct and learn hypergraph relationships from remote sensing features.

### 3.2. Overall Architecture

**Figure 2:**
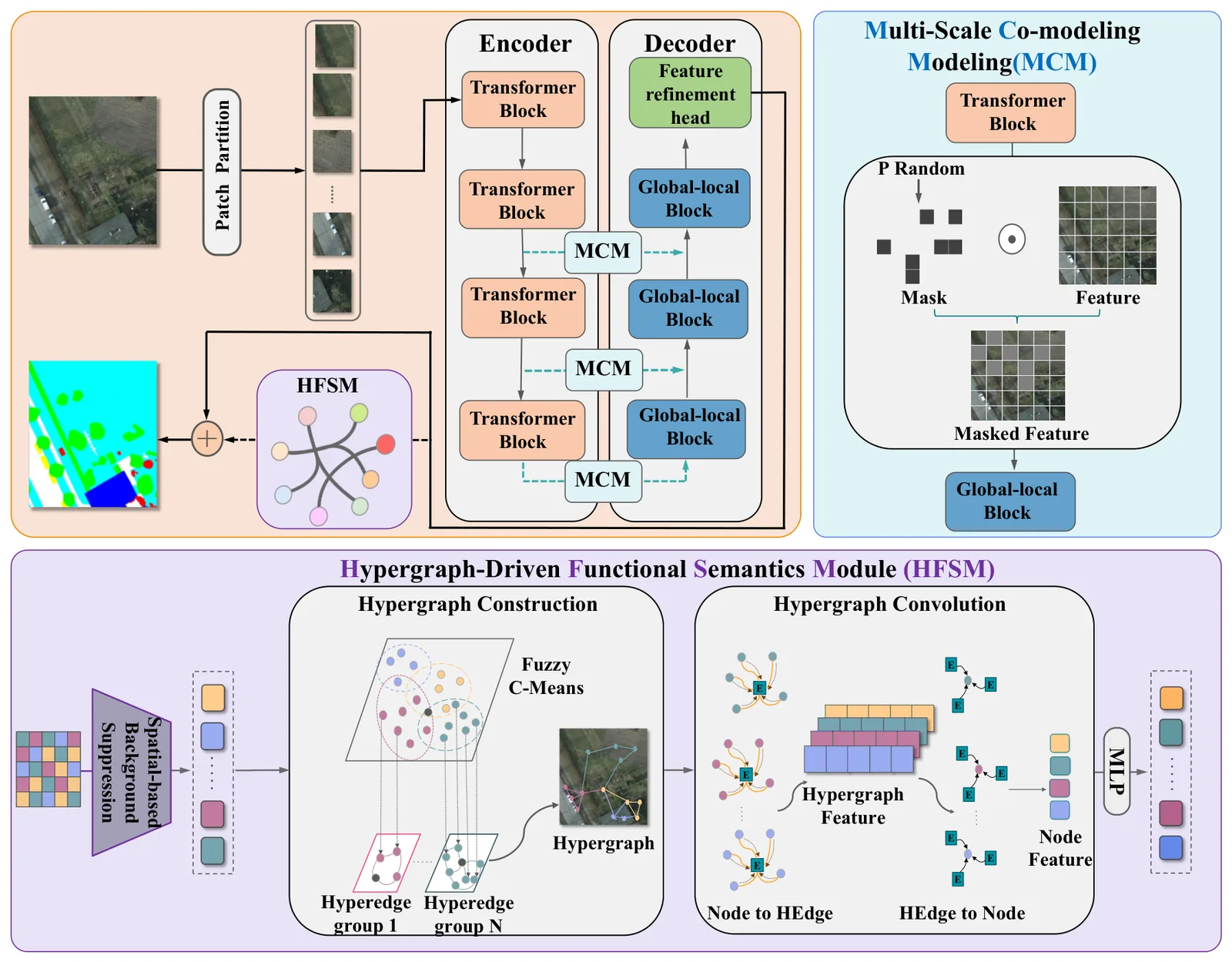
In our proposed HyperNet, the input image is first divided into multiple patches, which are then fed to a Transformer encoder. The encoder consists of four stages, each generating features at a different scale. Stochastic perturbations are then introduced via the MCM module. Subsequently, these features are processed by a decoder and passed to the HFSM module for hypergraph structure learning. Finally, a segmentation head produces the final segmentation map from the learned features.

Our proposed HyperNet architecture is illustrated in Figure 2. The proposed framework HyperNet addresses the limitations of static feature aggregation mechanisms in conventional approaches by integrating hypergraph-driven higher-order relation modeling into a hybrid Transformer-CNN architecture. Its overall structure is formulated in Equation (3) as:

(3) $$F=D\left(E(X)\right)\to H\left(F\right)\to L\left(Y_{\mathrm{pred}}\right),$$

where E denotes the hybrid encoder, D represents the multi-scale co-occurrence modeling decoder, H is the hypergraph-driven functional semantics module, and L stands for the hybrid loss function. The detailed design of each component is described below.

### 3.3. Hybrid Transformer–CNN Encoder

The encoder E employs a hierarchical architecture that balances global context capture with local detail preservation through window-based self-attention and cross-window interaction modules, as expressed in Equation (4):

(4) $$Z_{i}=\mathrm{Window}\text{-}\mathrm{MSA}\left(Z_{i-1}\right)+\mathrm{Cross}\text{-}\mathrm{Window}\text{-}\mathrm{Interaction}\left(Z_{i-1}\right),$$

where Window-MSA preserves fine-grained structures via self-attention within localized windows, while cross-window interaction aggregates dependencies across windows through a global–local dual-branch design. The resulting multi-scale feature maps ${\left\{F_{l}\right\}}_{l=1}^{L}$ encode a spatial hierarchy from coarse to fine, with exponentially decreasing spatial resolutions across layers.

### 3.4. Multi-Scale Co-Modeling Module

The decoder D facilitates dynamic multi-scale feature interaction through perturbation-driven feature refinement and adaptive fusion. For each encoder feature $F_{l}$, a stochastic mask $M_{l}=P\left(F_{l};p\right)$ is generated with a suppression ratio *p*:

(5) $$F_{\mathrm{masked},l}=F_{l}⊙M_{l},$$

where *p* controls the proportion of suppressed feature responses. The stochastic masking introduces controlled feature perturbations and reduces the model’s dependence on individual local activations.

The perturbed feature is then adaptively fused with the decoder feature $F_{\mathrm{dec}}$:

(6) $$F_{\mathrm{fuse},l}=\alpha ⊙F_{\mathrm{masked},l}+\left(1-\alpha \right)⊙F_{\mathrm{dec}}.$$

The fusion coefficient α is dynamically generated through a learnable gating mechanism:

(7) $$\alpha =\sigma \left(W_{\alpha }\;\mathrm{Concat}\left(F_{\mathrm{masked},l},F_{\mathrm{dec}}\right)\right),$$

where $W_{\alpha }$ denotes a learnable transformation and σ(·) is the sigmoid function. The resulting gating map α∈[0,1] adaptively controls the relative contributions of the perturbed encoder feature and the decoder feature. This design introduces controlled feature perturbations during training while preserving complementary contextual information through adaptive feature fusion.

### 3.5. Hypergraph-Driven Functional Semantics Module

To model higher-order spatial relationships, the functional semantics module H constructs a hypergraph G=(V,E,H). The node set $V=\{v_{1},v_{2},\ldots ,v_{N}\}$ consists of feature tokens extracted from the decoder output $F_{\mathrm{dec}}$, where each node $v_{i}\in R^{D}$ represents the semantic information of a spatial region.

We adopt fuzzy c-means (FCM) clustering to construct hyperedges. Unlike hard clustering, which assigns each node to a single cluster, FCM represents the association between a node and different clusters using continuous membership degrees. This property is consistent with the heterogeneous relationships in remote sensing scenes, where a spatial region may exhibit semantic associations with multiple surrounding regions rather than belonging to a single relational group.

Given node features $X={\left\{x_{i}\right\}}_{i=1}^{N}$, FCM minimizes the following objective:

(8) $$J_{\mathrm{FCM}}=\sum_{i=1}^{N}\sum_{m=1}^{M}\mu_{im}^{q}{∥x_{i}-c_{m}∥}_{2}^{2},$$

subject to

(9) $$\sum_{m=1}^{M}\mu_{im}=1,\;0\leq \mu_{im}\leq 1,$$

where $c_{m}$ denotes the center of the *m*-th cluster, $\mu_{im}$ denotes the membership degree of node $v_{i}$ to cluster *m*, and q>1 is the fuzziness coefficient.

The continuous membership $\mu_{im}$ provides a natural measure of the node–hyperedge association strength. A node can therefore contribute to multiple relational groups with different degrees, which avoids the exclusive assignment imposed by hard clustering. This property is particularly suitable for constructing weighted hyperedges from ambiguous and heterogeneous remote sensing features.

The hyperedge set $E=\{e_{1},e_{2},\ldots ,e_{M}\}$ is then constructed according to the FCM memberships. Only nodes satisfying $\mu_{im}\geq \tau$ are retained in hyperedge $e_{m}$:

(10) $$e_{m}=\left\{v_{i}|\mu_{im}\geq \tau \right\}.$$

In all experiments, the membership threshold τ is fixed at 0.1. This threshold filters weak node–hyperedge associations while allowing a node to participate in multiple hyperedges when meaningful soft memberships are retained. The same value is used across all datasets for consistent hypergraph construction.

The weighted incidence matrix $H\in R^{N\times M}$ is defined as

(11) $$H_{im}=\left\{\begin{array}{cc} \frac{\mu_{im}}{\sum_{j:\mu_{jm}\geq \tau }\mu_{jm}},\; & \mu_{im}\geq \tau ,\\ 0, & \mu_{im}<\tau . \end{array}\right.$$

where the normalized membership degree represents the relative association strength between node $v_{i}$ and hyperedge $e_{m}$.

Hypergraph convolution performs relational feature propagation in two stages of message passing. In the node-to-hyperedge aggregation phase, the feature matrix X is projected via a learnable weight matrix $W_{e}\in R^{D\times D}$ and aggregated to hyperedges using the transpose of H:

(12) $$Z_{e}=\mathrm{softmax}\left(H^{T}XW_{e}\right),$$

which maps multi-node features into hyperedge-level semantic representations. Subsequently, during the hyperedge-to-node diffusion phase, hyperedge features $Z_{e}$ are propagated back to nodes via a learnable weight matrix $W_{v}\in R^{D\times D}$:

(13) $$Z_{v}=\mathrm{softmax}\left(HZ_{e}W_{v}\right).$$

where $W_{v}$ enforces consistency among hyperedge features. The final relational embedding is obtained through a residual connection:

(14) $$Z^{\prime }=Z_{v}+X,$$

which preserves the original node features while incorporating higher-order relational information. The resulting representation is then used for downstream segmentation.

### 3.6. Hybrid Loss Function

The loss function L combines Softmax cross-entropy ($L_{ce}$) and Dice loss ($L_{dice}$). Cross-entropy loss provides pixel-wise classification supervision, whereas Dice loss encourages region-level overlap. The two losses are defined as

(15) $$\begin{array}{cc} L_{ce} & =-\sum_{i=1}^{N}\sum_{k=1}^{K}y_{ik}\log{\hat{y}}_{ik},\\ L_{dice} & =1-\frac{1}{K}\sum_{k=1}^{K}\frac{2\sum_{i=1}^{N}{\hat{y}}_{ik}y_{ik}}{\sum_{i=1}^{N}\left({\hat{y}}_{ik}^{2}+y_{ik}^{2}\right)},\\ L & =L_{ce}+\lambda L_{dice}, \end{array}$$

where *N* and *K* denote the number of pixels and semantic classes, respectively. The weighting coefficient λ is set to 1.5 to balance the contributions of the two loss terms.

## 4. Datasets

We evaluate HyperNet on three widely used remote sensing semantic segmentation benchmarks, including the ISPRS Vaihingen dataset, the ISPRS Potsdam dataset, and LoveDA. These datasets cover different geographical regions, spatial resolutions, and scene characteristics. Their main imaging properties are summarized in Table 1.

### 4.1. Vaihingen Dataset

The ISPRS Vaihingen dataset consists of 33 very-high-resolution true orthophoto (TOP) tiles acquired over Vaihingen an der Enz, Germany. The tiles have varying spatial sizes, with an average size of approximately 2494×2064 pixels, and a ground sampling distance (GSD) of 0.09 m. The TOP images are provided as three-band images corresponding to near-infrared (NIR), red, and green. Although the benchmark also provides digital surface model (DSM) data, only the three optical TOP bands are used in our experiments.

The semantic annotations contain six categories: impervious surfaces, buildings, low vegetation, trees, cars, and clutter/background.

### 4.2. Potsdam Dataset

The ISPRS Potsdam dataset contains 38 very-high-resolution TOP tiles acquired over Potsdam, Germany. Each tile has a spatial size of 6000×6000 pixels with a GSD of 0.05 m. The benchmark provides the TOP imagery in RGB, IRRG, and RGBIR channel compositions, together with DSM data. In this study, only the RGB bands are used, without incorporating DSM or normalized DSM information. The semantic categories are identical to those of the Vaihingen dataset.

### 4.3. LoveDA Dataset

LoveDA is a high-spatial-resolution land-cover dataset containing 5987 remote sensing images collected from urban and rural areas of Nanjing, Changzhou, and Wuhan, China. The images were obtained from the Google Earth platform and have a GSD of 0.3 m and a spatial size of 1024×1024 pixels. The released images are three-channel RGB images. The dataset contains seven semantic categories: background, building, road, water, barren land, forest, and agriculture.

We follow the official training-validation-test split, consisting of 2522 training images, 1669 validation images, and 1796 test images. The inclusion of both urban and rural scenes introduces substantial variations in object scale, background appearance, and class distribution.

The exact wavelength ranges or spectral response functions of the released benchmark images are not specified in the official dataset documentation. Therefore, Table 1 reports the spectral bands actually used in our experiments rather than assigning approximate wavelength intervals that are not provided by the dataset sources.

The Vaihingen and Potsdam images are distributed as true orthophoto products, indicating that geometric and orthorectification processing has been performed during dataset generation. However, the official benchmark documentation does not explicitly specify whether atmospheric correction was applied to the released image products. Atmospheric correction is likewise not explicitly documented for the released LoveDA images. We therefore used the officially released benchmark images directly and did not perform any additional atmospheric correction.

## 5. Experiments

### 5.1. Evaluation Metrics

We adopted the overall accuracy (OA), mean F1 Score (F1), and mean intersection over union (mIoU) as the evaluation metrics. For LoveDA, only mIoU is reported following its standard evaluation protocol. The model is trained using only these datasets, without any additional dataset for supervised training.

### 5.2. Implementation Details

To ensure a fair comparison with other methods, we followed the settings used in previous methods. For the Vaihingen dataset, we exclusively utilized the optical spectral bands of TOP imagery, excluding DSM/NDSM data. The dataset was divided into 17 test tiles (IDs: 2, 4, 6, 8, 10, 12, 14, 16, 20, 22, 24, 27, 29, 31, 33, 35, 38) and 16 training tiles. For the Potsdam dataset, we selected 14 test tiles (IDs: 2_13, 2_14, 3_13, 3_14, 4_13, 4_14, 4_15, 5_13, 5_14, 5_15, 6_13, 6_14, 6_15, 7_13) and 24 training tiles. The listed IDs are the official identifiers of spatially distinct orthophoto tiles in the ISPRS benchmarks and are used only to specify the training/test split. They do not represent individual surface types; each tile contains multiple land-cover classes, including impervious surfaces, buildings, low vegetation, trees, cars, and clutter/background. LoveDA follows the official training-validation-test split. All original tiles were cropped into 1024×1024 patches. During training, the input images were randomly cropped into 512×512 patches. Data augmentation included random scaling with factors of {0.5,0.75,1.0,1.25,1.5}, random vertical flipping, random horizontal flipping, and random rotation.

All models were trained for 80 epochs with a batch size of 2 using the AdamW optimizer. The initial learning rate was set to $6\times 10^{-4}$ for Vaihingen and $5\times 10^{-4}$ for LoveDA and Potsdam. The weight decay was set to $2.5\times 10^{-4}$ for Vaihingen and Potsdam and 0.01 for LoveDA. The backbone weight decay was set to the same value as the corresponding weight decay. No learning-rate warmup strategy was used. The learning rate was adjusted using the CosineAnnealingWarmRestarts scheduler with $T_{0}=15$ and $T_{\mathrm{mult}}=2$. The number of data-loading workers was set to 4. The main comparison experiments and key component ablation experiments were independently repeated three times using random seeds 42, 43, and 44. The corresponding results are reported as mean ± standard deviation.

During inference, each full-resolution test tile is first padded along the bottom and right boundaries so that its spatial dimensions are divisible by the patch size. The padded tile is then partitioned into non-overlapping 512×512 patches with a stride of 512 pixels. Each patch is processed independently, and the predictions are stitched back to their original spatial locations and cropped to the original tile size. Therefore, inference is performed using fixed-size, non-overlapping grid patches rather than feeding the entire tile into the network in a single forward pass. The same test-time flip augmentation settings were applied to HyperNet, the internally evaluated FT-UNetFormer baseline, and all ablation configurations. The results of the other state-of-the-art methods in the comparison tables were taken from their original publications; therefore, their test-time augmentation settings follow the corresponding original implementations and were not controlled by us.

In the HFSM module, the ratio of selected patch tokens *a* in spatial-based background suppression and the number of node clusters *b* for hypergraph construction were set to 0.3 and 5, respectively. The membership threshold τ for hyperedge construction was fixed at 0.1. In the MCM, the perturbation ratio *p* for each stage was set to 0.1. All experiments were conducted using PyTorch 1.8.1 on NVIDIA GeForce RTX 2080 Ti GPUs (NVIDIA Corporation, Santa Clara, CA, USA). To facilitate reproducibility, the source code, training and evaluation configurations, and trained model checkpoints will be publicly released upon publication.

### 5.3. Comparison with State-of-the-Art Methods

We compare the proposed HyperNet with existing state-of-the-art CNN-based and Transformer-based methods on the LoveDA, Vaihingen, and Potsdam datasets. The results of HyperNet are reported as mean ± standard deviation over three independent runs with random seeds 42, 43, and 44, while the results of the compared methods are taken from their original publications.

**LoveDA:** Table 2 compares HyperNet with existing CNN-based and Transformer-based methods. HyperNet achieves an mIoU of 54.70±0.12%, obtaining state-of-the-art performance on the LoveDA dataset. Compared with UNetFormer and the best existing result of ViT-G12X4, HyperNet achieves numerical mIoU improvements of 2.30% and 0.30%, respectively. These results demonstrate the effectiveness of modeling heterogeneous spatial relationships in complex remote sensing scenes.
**Vaihingen:** As shown in Table 3, HyperNet achieves an F1 score of 91.70±0.18%, an mIoU of 85.01±0.11%, and an OA of 93.50±0.06%. In particular, HyperNet improves the mIoU from 84.10% for FT-UNetFormer to 85.01%, corresponding to an improvement of 0.91%. It also achieves the highest mIoU among the compared methods. The improvement indicates that incorporating higher-order heterogeneous spatial relationship modeling into the baseline provides additional benefits for remote sensing segmentation.
**Potsdam:** Table 4 compares HyperNet with existing CNN-based and Transformer-based methods on the Potsdam dataset. HyperNet achieves an F1 score of 93.29±0.05%, an mIoU of 87.64±0.08%, and an OA of 92.13±0.04%.

**Table 2:** Comparison experiments with other state-of-the-art methods on LoveDA dataset.

Method	Model	Year	mIoU
HRNet32 [34]	CNN	2021	49.79
LSKNet-S [35]	CNN	2023	54.00
LSKNet-T [35]	CNN	2023	53.20
Hi-ResNet [36]	CNN	2024	52.60
LWGANet L2 [37]	CNN	2025	53.60
CM-UNet [15]	CNN	2024	52.17
LOGCAN++ [20]	CNN	2025	53.35
UNetFormer [8]	Transformer	2021	52.40
ViT-B+RVSA-UperNet [38]	Transformer	2022	51.95
VITAE-BRVSA-UperNet [38]	Transformer	2022	52.44
ViT-G12X4 [39]	Transformer	2023	54.40
AerialFormer [40]	Transformer	2023	54.10
SelectiveMAE+ViT-L [41]	Transformer	2024	54.31
MAE+MTP(ViT-L+RVSA) [42]	Transformer	2024	54.17
IMP+MTP(internimage-XL) [42]	Transformer	2024	54.17
DecoupleNet D2 [43]	Transformer	2024	53.10
MAE+MTP(ViT-B+RVSA) [42]	Transformer	2024	52.39
HyperNet (Ours)	Transformer	2025	**54.70 ± 0.12**

*Note:* Bold indicates the best result.

Compared with LSKNet-S, which achieves the highest mIoU among the CNN-based methods in Table 4, HyperNet improves the mIoU from 87.20% to 87.64%, corresponding to a numerical improvement of 0.44%. Compared with FT-UNetFormer, HyperNet obtains numerical improvements of 0.14% in mIoU and 0.13% in OA, while its F1 score is 0.01% lower. Compared with DC-Swin, HyperNet improves F1, mIoU, and OA by 0.04%, 0.08%, and 0.13%, respectively. In addition, HyperNet improves the mIoU over PDSSNet by 0.09%.

**Table 3:** Comparison experiments with other state-of-the-art methods on the Vaihingen dataset.

Method	Model	Year	F1	mIoU	OA
DANet [17]	CNN	2019	79.60	69.40	88.20
ABCNet [44]	CNN	2021	-	-	90.70
MANet [45]	CNN	2021	-	-	90.96
CMTFNet [46]	CNN	2023	87.42	77.95	88.71
UPerNet(SAP) [47]	CNN	2024	-	73.27	90.1
ESDINet [48]	CNN	2024	89.99	82.03	90.88
DPFE [49]	CNN	2024	-	-	91.30
RS3Mamba [50]	CNN	2024	90.70	83.30	93.20
BANet [51]	Transformer	2021	-	-	90.5
DC-Swin [52]	Transformer	2021	90.7	-	91.6
Segmenter [25]	Transformer	2021	84.10	73.60	88.10
UNetFormer [8]	Transformer	2021	90.4	82.7	91.0
FT-UNetFormer [8]	Transformer	2021	91.32	84.10	91.62
SFA-Net [53]	Transformer	2024	91.20	-	-
HyperNet (Ours)	Transformer	2025	**91.70 ± 0.18**	**85.01 ± 0.11**	**93.50 ± 0.06**

*Note:* The best results are highlighted in bold.

**Table 4:** Comparison experiments with other state-of-the-art methods on the Potsdam dataset.

Method	Model	Year	F1	mIoU	OA
MAResU-Net [54]	CNN	2022	91.89	85.22	90.28
MANet [45]	CNN	2021	-	-	91.32
ABCNet [44]	CNN	2021	-	-	91.30
PSPNet(SAP) [47]	CNN	2024	-	74.30	88.56
LSKNet-S [35]	CNN	2024	93.10	87.20	92.00
DPFE [49]	CNN	2024	-	-	89.70
BANet [51]	Transformer	2021	-	-	91.06
UNetFormer [8]	Transformer	2021	92.80	86.80	91.30
FT-UNetFormer [8]	Transformer	2021	93.30	87.50	92.00
DC-Swin [52]	Transformer	2021	93.25	87.56	92.00
Mask2Former [55]	Transformer	2022	92.37	86.03	90.83
MPCNet [56]	Transformer	2023	92.29	85.91	90.56
RS-Mamba [57]	Transformer	2024	90.50	82.87	90.98
PyMamba [58]	Transformer	2024	90.30	82.52	90.91
Samba [59]	Transformer	2024	90.42	82.75	90.99
SFA-Net [53]	Transformer	2024	**93.50**	-	-
PDSSNet [60]	Transformer	2025	93.25	87.55	91.84
HyperNet (Ours)	Transformer	2025	93.29 ± 0.05	**87.64 ± 0.08**	**92.13 ± 0.04**

*Note:* The best results are highlighted in bold.

We also note that SFA-Net achieves a higher F1 score of 93.50%, exceeding HyperNet by 0.21%. Therefore, HyperNet does not achieve the best result on every evaluation metric. Nevertheless, it achieves the highest reported mIoU and OA among the compared methods while maintaining a competitive F1 score.

**Class-wise error analysis.** To further investigate the remaining segmentation errors, we compare the per-class performance of HyperNet with the FT-UNetFormer baseline on the Vaihingen dataset, as shown in Table 5. Among all categories, Low Vegetation exhibits the lowest performance, with an IoU of 73.19% and an F1 score of 84.52%, followed by Tree with an IoU of 81.94% and an F1 score of 90.07%. Compared with the baseline, the IoU of Low Vegetation and Tree decreases by 1.81% and 1.21%, respectively, indicating that vegetation-related categories remain the main source of segmentation errors.

In contrast, HyperNet improves the IoU of Impervious Surfaces, Buildings, and Cars by 6.44%, 0.17%, and 0.41%, respectively. In particular, the substantial improvement on Impervious Surfaces indicates that the proposed heterogeneous spatial relationship modeling is effective for large-scale structured regions. The relatively lower performance on Low Vegetation and Tree indicates that distinguishing different vegetation categories remains a limitation of the current model.

### 5.4. Ablation Experiments

We perform ablation experiments to evaluate the contributions of different components in HyperNet and test the effects of various hyperparameter settings on the final segmentation performance.

**Effectiveness of key components.** We conduct ablation studies to evaluate the individual contributions of HFSM and MCM, as shown in Table 6. Compared with the baseline, introducing HFSM improves the mIoU by 0.71%, while MCM yields an improvement of 0.52%. Although the individual gains are moderate, both modules consistently improve the segmentation performance over the strong baseline. When HFSM and MCM are jointly employed, the mIoU improvement reaches 0.91%. More specifically, adding MCM on top of HFSM further improves the F1, mIoU, and OA by 0.07%, 0.20%, and 0.03%, respectively. Given the relatively small magnitude of these marginal differences, we interpret them as numerical improvements rather than evidence of statistically significant gains. HFSM provides the larger individual gain by introducing higher-order heterogeneous spatial relationship modeling, whereas MCM provides a complementary benefit through robust multi-scale feature interaction.

To further evaluate the generalizability of the two modules, we repeat the component ablation study on the LoveDA dataset. As shown in Table 7, HFSM and MCM individually improve the mIoU by 0.48% and 0.34%, respectively, while their combination yields an overall improvement of 0.86%. The consistent improvement trends on both Vaihingen and LoveDA indicate that HFSM and MCM contribute from different perspectives and that their effectiveness is not limited to a single dataset.

**Figure 3:**
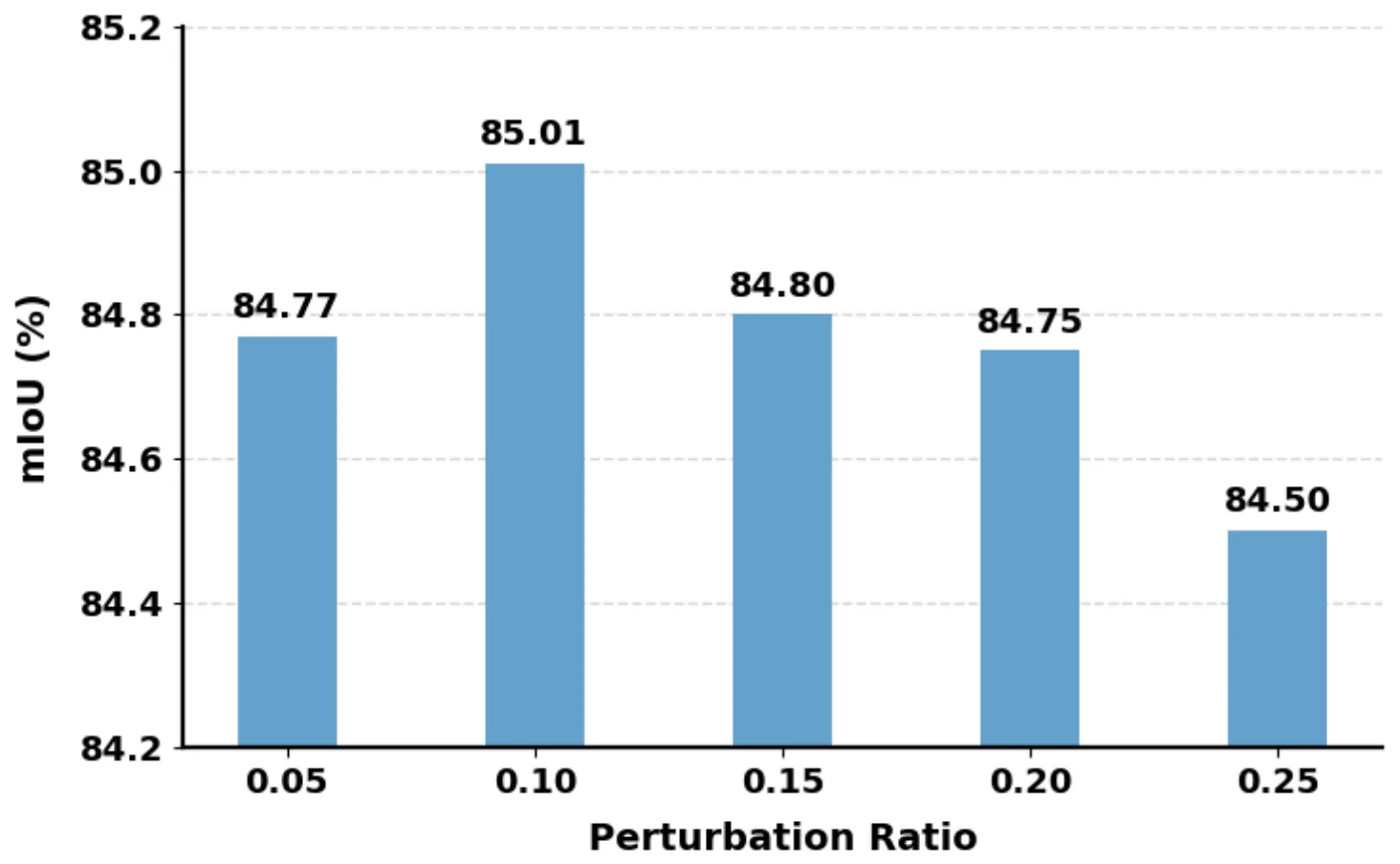
Evaluation of different ratios of the perturbation strategy on the Vaihingen dataset.

**Figure 4:**
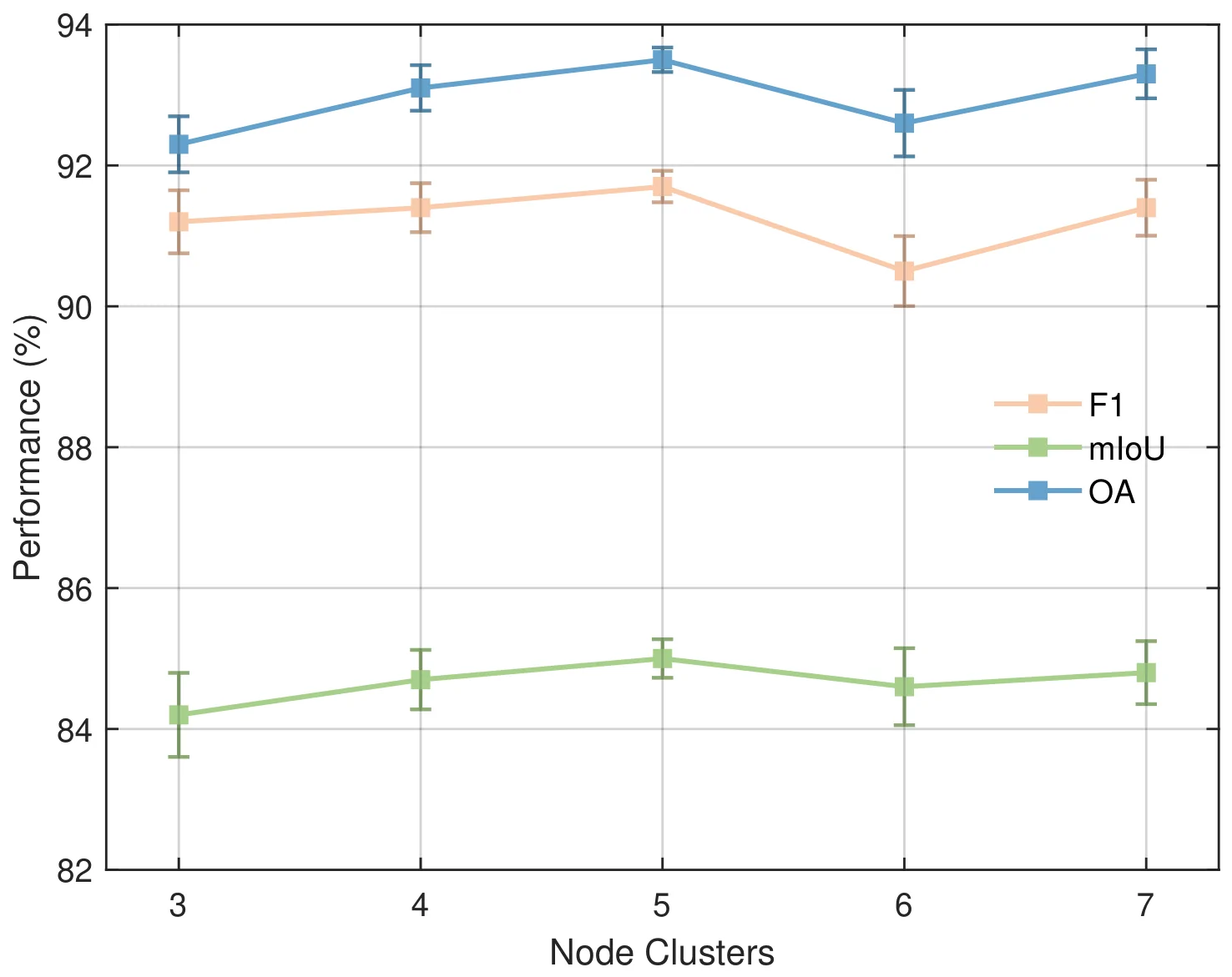
Evaluation of different clusters on the Vaihingen dataset.

**Impact of the ratio of perturbation strategy.** The experimental results in Figure 3 indicate that a perturbation ratio of 0.1 yields the optimal segmentation performance on the Vaihingen dataset. This phenomenon can be explained by the balance between feature diversity augmentation and semantic consistency preservation in remote sensing (RS) image segmentation. RS images are characterized by complex spatial layouts and fine-grained semantic details (e.g., small-scale objects like vehicles adjacent to large buildings). A perturbation ratio that is too low fails to introduce sufficient diversity in scale variations. In such cases, the model is exposed to limited perturbations during training, leading to overfitting to the original scale distribution of the dataset. Consequently, it struggles to generalize to unseen scale variations in test data (e.g., objects captured at slightly different resolutions), resulting in degraded robustness. Conversely, a perturbation ratio that is too high disrupts the inherent semantic consistency of RS features. For instance, excessive perturbations may distort the positional relationships between correlated objects (e.g., roads connecting buildings) or blur critical boundary information (e.g., the edge between vegetation and bare soil). This destruction of semantic integrity misleads the model during training, as it learns noisy or irrelevant scale-dependent patterns, ultimately reducing segmentation accuracy. Thus, a ratio of 0.1 strikes a critical balance. It introduces moderate scale perturbations to simulate real-world variations while preserving the core semantic structure of RS scenes. This enables the model to learn both robust scale-invariant features and accurate spatial relationships.
**Impact of the number of node clusters.** Figure 4 evaluates the effect of the number of node clusters *b* on the Vaihingen dataset. Each configuration was independently repeated three times, and the results are reported as mean ± standard deviation. Among the evaluated settings, b=5 achieves the highest mean performance and is therefore adopted in the remaining experiments.

The results suggest that the number of clusters affects the granularity of hypergraph construction. When the number of clusters is relatively small, semantically different feature nodes may be grouped into coarse hyperedges, which can weaken the discrimination of heterogeneous spatial relationships. In contrast, increasing the number of clusters produces finer partitions and may fragment semantically related nodes into different hyperedges. Therefore, an intermediate number of clusters can provide a better balance between coarse relational grouping and excessive hyperedge fragmentation.

It is also worth noting that the performance does not vary monotonically with *b*. In particular, a noticeable decrease is observed at b=6, followed by a partial recovery at b=7. Changing *b* causes a discrete reorganization of fuzzy cluster memberships rather than a continuous refinement of the previous partition. Consequently, different values of *b* can produce different hyperedge compositions and higher-order information propagation patterns. This non-monotonic behavior indicates that the model exhibits some sensitivity to the cluster configuration. Nevertheless, among the evaluated discrete settings, b=5 consistently provides the highest mean segmentation performance and is selected as the default configuration.

**Impact of different clustering algorithms.** Table 8 compares different strategies for constructing the hypergraph on the Vaihingen dataset. Among them, FCM achieves the best overall performance, with F1, mIoU, and OA scores of 91.70%, 85.01%, and 93.50%, respectively.

**Table 8:** Comparison of different clustering algorithms on the Vaihingen dataset.

Clustering Algorithm	F1 (%)	mIoU (%)	OA (%)
K-Nearest Neighbors	91.32	84.52	93.18
Spectral Clustering	91.38	84.61	93.32
K-Means Clustering	91.53	84.63	92.62
Fuzzy C-Means	**91.70**	**85.01**	**93.50**

*Note:* The best results are highlighted in bold.

Different grouping strategies characterize feature relationships in different ways. K-Nearest Neighbors constructs local associations according to feature similarity, which is effective for capturing neighborhood relationships but may provide limited flexibility for modeling nodes with ambiguous associations. Spectral Clustering exploits the similarity structure of the feature space and can capture non-linear grouping patterns, but ultimately produces discrete cluster assignments. K-Means partitions feature nodes according to their distances to cluster centers, providing a simple and efficient grouping strategy, but each node is assigned to only one cluster.

In contrast, FCM assigns each feature node a continuous membership degree to multiple clusters. Such soft assignments naturally correspond to the association strengths between nodes and hyperedges, allowing one node to participate in multiple relational groups with different weights. This property is particularly suitable for heterogeneous remote sensing feature representations, where the associations among spatial regions may not always be strictly exclusive. Therefore, FCM provides more flexible node–hyperedge associations and achieves the best overall performance among the evaluated strategies. It is also worth noting that OA does not always follow the same trend as mean F1 and mIoU. For example, K-Means achieves the second-best F1 and mIoU scores of 91.53% and 84.63%, respectively, but obtains a lower OA of 92.62%. This difference results from the distinct weighting characteristics of these metrics. OA is calculated over all pixels and is therefore more sensitive to performance changes in classes occupying larger pixel proportions, whereas mean F1 and mIoU average class-wise performance and assign equal importance to individual semantic classes. Consequently, different distributions of class-wise errors can lead to different rankings between OA and the class-averaged metrics.

**Impact of the loss weighting coefficient.** We further evaluate the influence of the weighting coefficient λ in the hybrid loss function. As shown in Table 9, the model achieves the best performance when λ = 1.5, with an mIoU of 85.01%. Increasing λ from 0.5 to 1.5 gradually improves the segmentation performance, indicating that an appropriate contribution of Dice loss benefits region-level prediction. However, further increasing λ leads to a slight performance decrease. This suggests that an excessively large Dice weight may weaken the balance between pixel-wise classification and region-level supervision. Therefore, λ = 1.5 is adopted in all experiments.
**Computational complexity analysis.** To quantify the computational overhead introduced by the proposed modules, we compare the baseline, HFSM-only, MCM-only, and full HyperNet in terms of parameter count, FLOPs, inference latency, peak inference memory, and mIoU, as shown in Table 10. FLOPs, inference latency, and peak inference memory are measured using 512×512 input images under the same inference setting.

Compared with the baseline, the full HyperNet increases the parameter count from 95.98 M to 107.47 M and the FLOPs from 507.19 G to 559.66 G, corresponding to increases of approximately 12.0% and 10.3%, respectively. The inference latency increases from 137.06 ms to 181.20 ms per image, while the peak inference memory increases from 0.88 GB to 1.29 GB. In return, the mIoU improves from 84.10% to 85.01%.

The module-wise comparison further shows that HFSM accounts for most of the additional computational cost due to fuzzy clustering, hypergraph construction, and node–hyperedge message propagation. In contrast, MCM introduces relatively limited overhead, increasing the parameter count from 95.98 M to 96.03 M and the FLOPs from 507.19 G to 508.12 G, while improving the mIoU from 84.10% to 84.62%. These results provide a quantitative characterization of the accuracy–efficiency trade-off of HyperNet.

**Figure 5:**
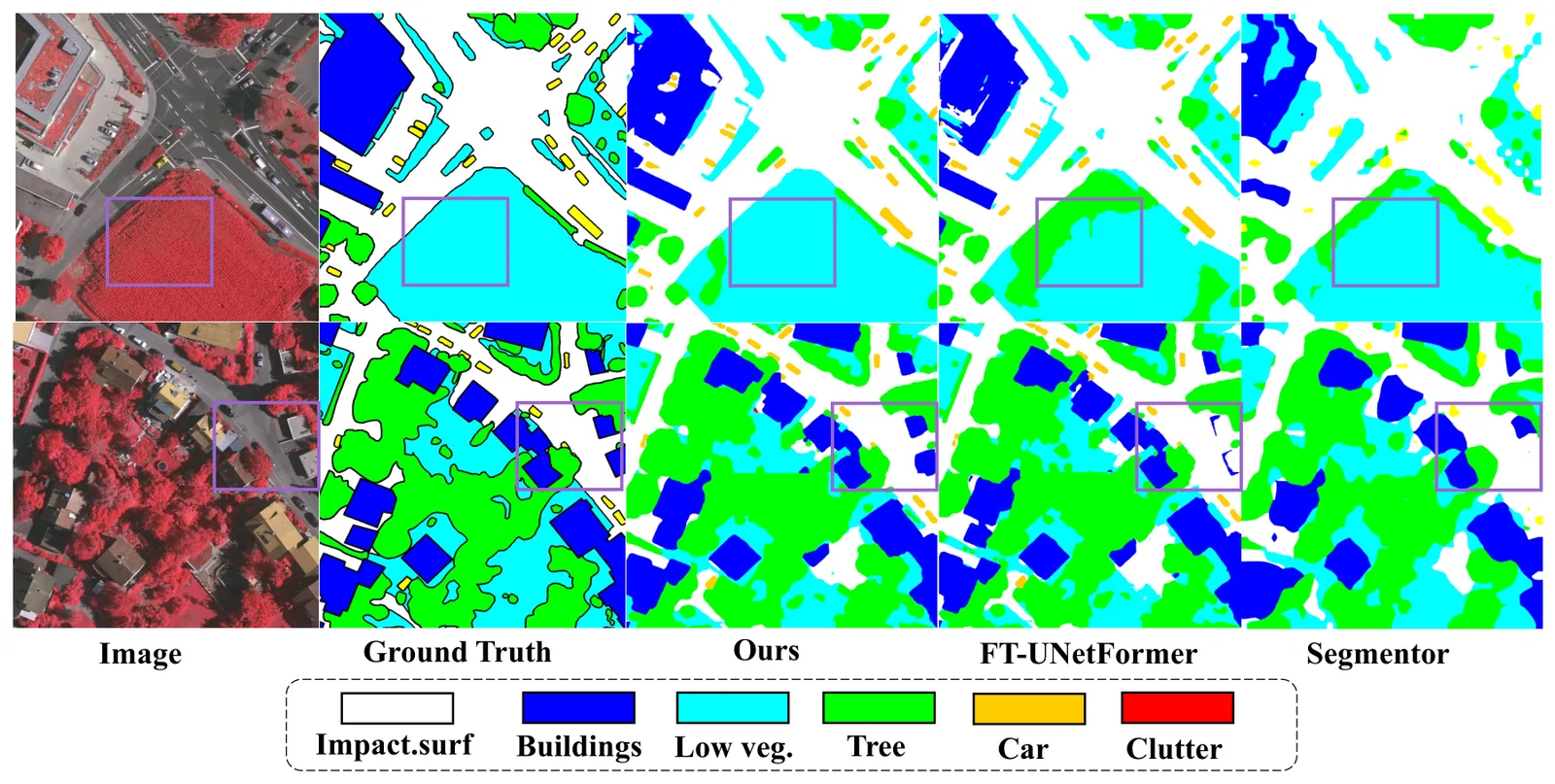
Visualization results of IDs 10 and 14 from the Vaihingen test set. The first column denotes the input NIR–R–G TOP images, the second column shows the ground truth, and the remaining columns show the segmentation results of different methods. The purple boxes highlight representative regions for qualitative comparison.

**Figure 6:**
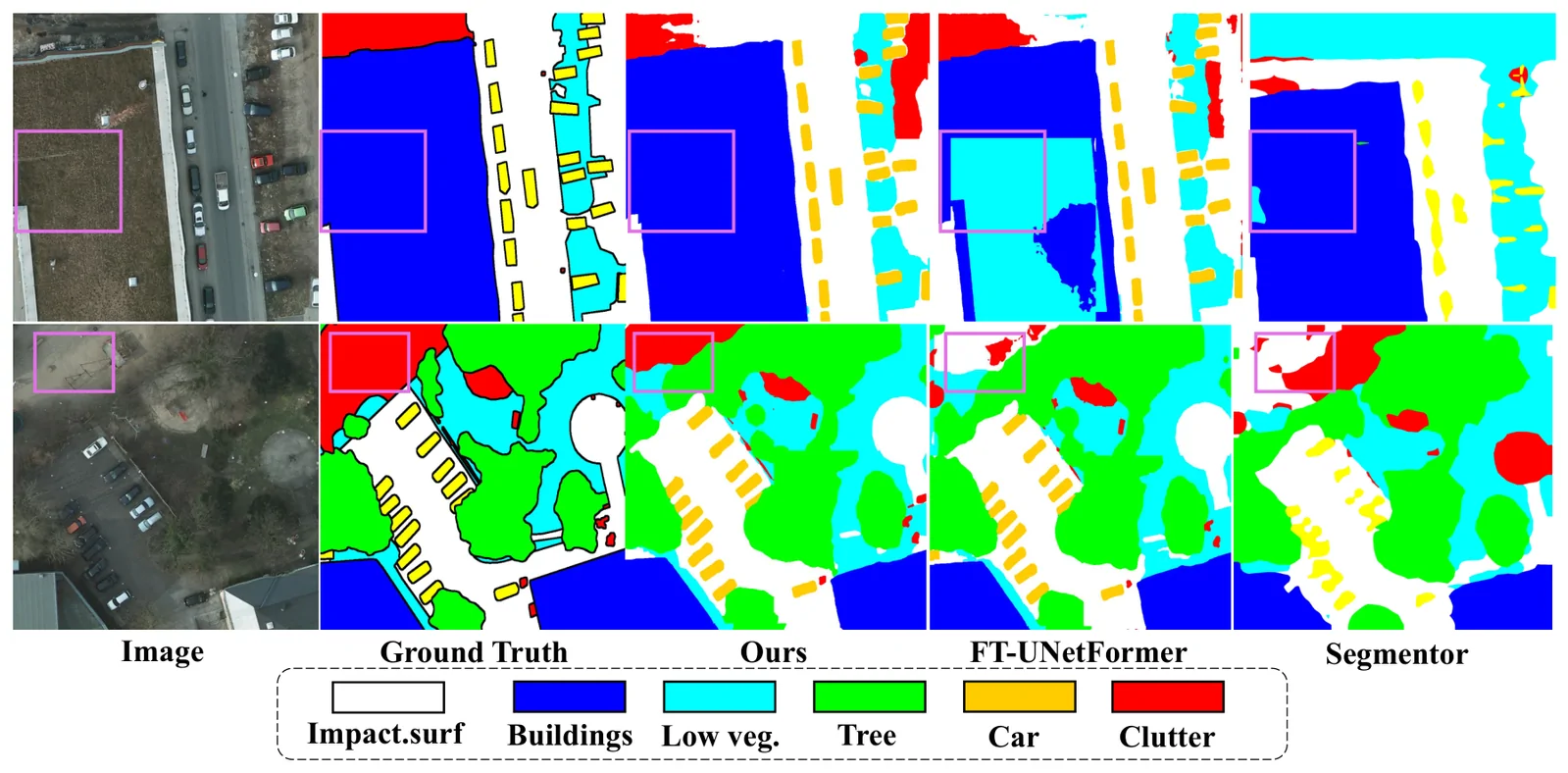
Visualization results of ID 4_13 and 6_15 from the Potsdam test set. The first column denotes the input RGB images. The third column shows the segmentation results of the proposed HyperNet. The purple boxes highlight representative regions for qualitative comparison.

**Figure 7:**
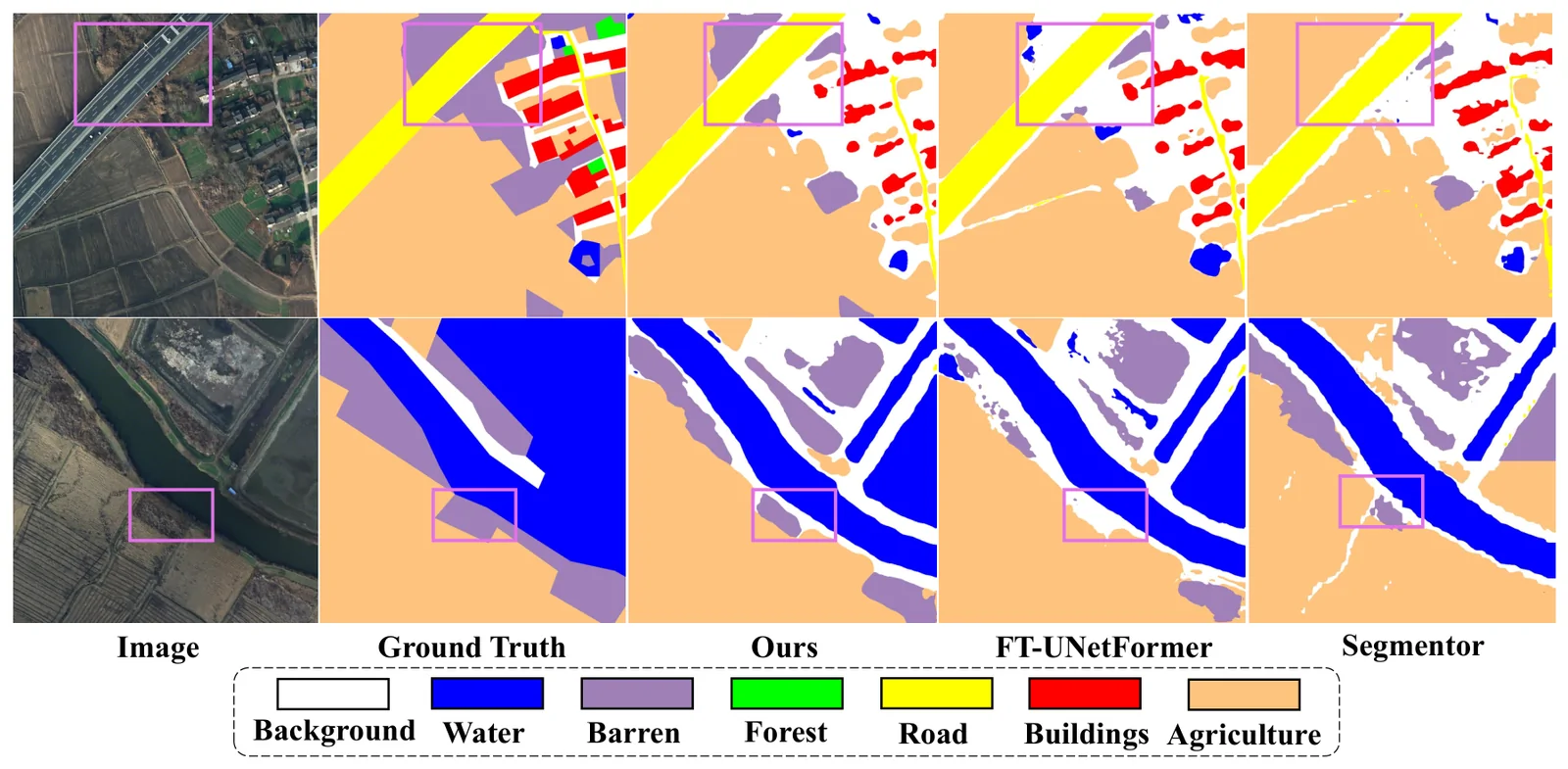
Visualization results of ID 2558 and 2585 from the LoveDA test set. The first column denotes the input RGB images. The third column shows the segmentation results of the proposed HyperNet. The purple boxes highlight representative regions for qualitative comparison.

**Visualization.** We further present qualitative comparisons of our method across the three employed datasets in Figure 5, Figure 6 and Figure 7.

By integrating the hypergraph-perception module we developed, the model effectively augments the feature embedding with rich, multi-faceted spatial relationships among entities, thereby strengthening inter-entity coherence. Compared to the baseline FT-UNetFormer [8], our newly designed multi-scale modeling component perturbs features at each scale in a targeted manner. This encourages the network to distinguish between disparate resolutions and produce more precise boundaries—even when the scene contains objects of varying sizes (such as buildings and vehicles). The qualitative results show that HyperNet produces more coherent predictions in scenes containing objects with different scales and spatial layouts. These observations are consistent with the intended roles of MCM in improving cross-scale feature interaction and HFSM in enhancing higher-order contextual information propagation.

## 6. Discussion

**Figure 8:**
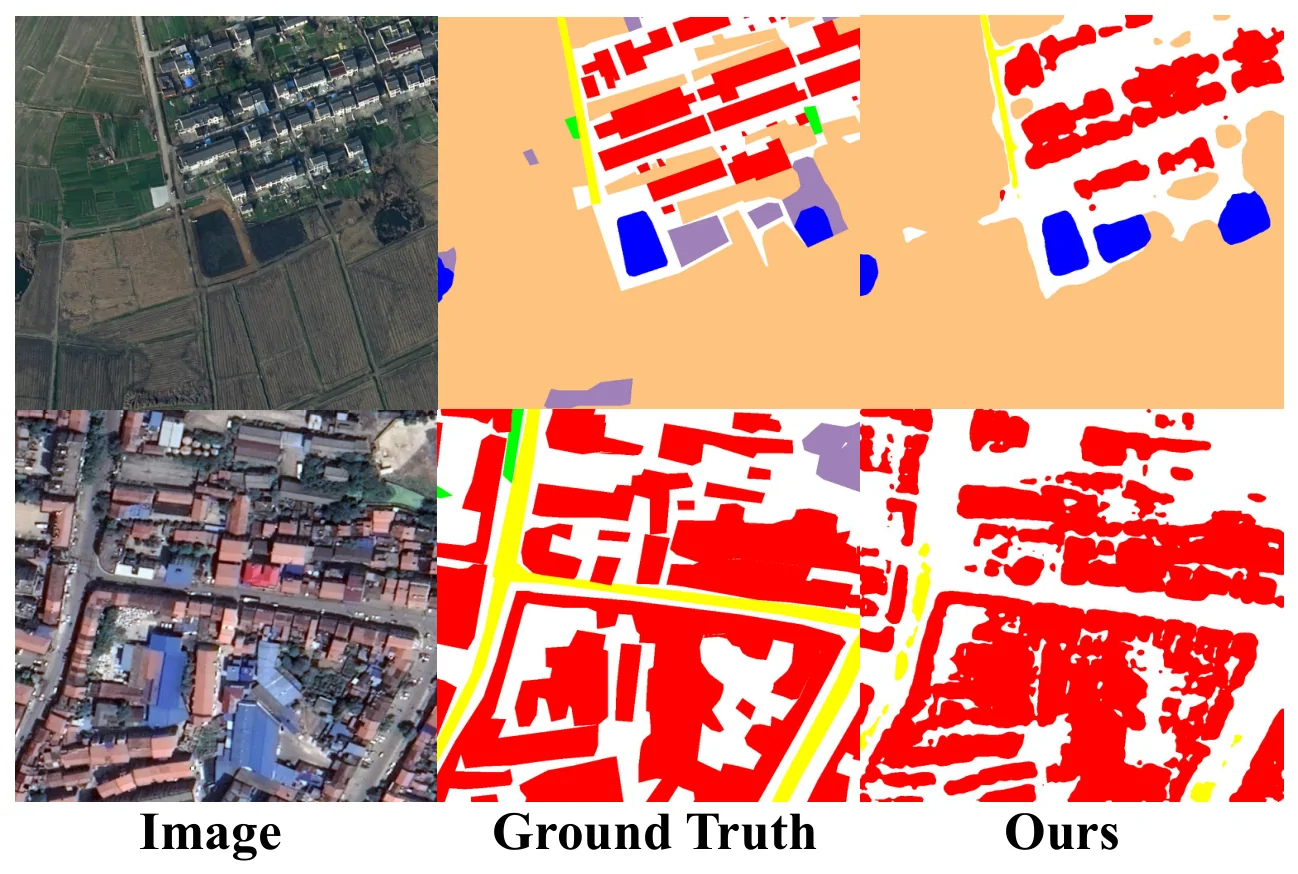
Representative failure cases on the LoveDA dataset. The colors correspond to the semantic classes and follow the same color scheme as in Figure 7.

In addition to the quantitative comparisons, the class-wise and failure-case analyses provide further insights into the behavior of HyperNet. As shown in Figure 8, segmentation errors mainly occur in low-quality regions with blurred boundaries and in scenes containing small or densely distributed objects. These observations are consistent with the class-wise results, where vegetation-related categories remain relatively challenging. Together with the quantitative experiments, these results reveal both the strengths and remaining limitations of the proposed higher-order relational modeling strategy.

The experimental results across LoveDA, Vaihingen, and Potsdam demonstrate that HyperNet achieves competitive segmentation performance across different remote sensing scenes. The ablation experiments further show that both HFSM and MCM contribute to the performance improvement over the baseline. HFSM provides the larger individual improvement, while MCM offers a complementary contribution when combined with HFSM. Nevertheless, the magnitude of the improvement varies across datasets and evaluation metrics. In particular, on the Potsdam dataset, SFA-Net achieves a higher F1 score of 93.50% than the 93.29±0.05% obtained by HyperNet, whereas HyperNet achieves the highest reported mIoU and OA among the compared methods. Therefore, the experimental results support the effectiveness of the proposed modules, but do not indicate uniform superiority over existing methods across all evaluation criteria.

The experiments also reveal that different evaluation metrics may respond differently to the same model configuration. In the clustering comparison, K-Means achieves competitive mean F1 and mIoU but a relatively lower OA. A similar behavior is observed for the MCM-only configuration, which obtains competitive mean F1 and mIoU while showing a lower OA than the HFSM-only configuration. This discrepancy is consistent with the different weighting mechanisms of these metrics. OA is calculated over all pixels and is therefore more strongly influenced by classes occupying larger pixel proportions, whereas mean F1 and mIoU average class-wise performance and assign equal importance to individual semantic classes. The class-wise analysis on the Vaihingen dataset further shows that the performance changes are not uniform across categories. HyperNet improves the segmentation of Impervious Surfaces, Buildings, and Cars, while lower performance is observed for Low Vegetation and Trees relative to the baseline. These results indicate that the performance of a segmentation model should be assessed jointly using multiple metrics and class-wise behavior rather than relying on a single aggregate measure.

The node-cluster sensitivity analysis provides another important observation. The segmentation performance does not vary monotonically with the number of clusters. Although b=5 provides the best overall mean performance among the evaluated settings, a noticeable decrease is observed at b=6, followed by a partial recovery at b=7. Changing *b* does not continuously refine a fixed partition; instead, it causes a discrete reorganization of fuzzy memberships and consequently changes the composition of the constructed hyperedges. Different cluster configurations can therefore lead to different higher-order information propagation patterns. This behavior indicates that the current hypergraph construction retains some sensitivity to the cluster configuration and that the selected value of b=5 should be regarded as the best-performing configuration among the evaluated settings rather than a universally optimal value.

The computational analysis further clarifies the accuracy–efficiency trade-off introduced by the proposed modules. Compared with the baseline, the full HyperNet increases the parameter count from 95.98 M to 107.47 M and the FLOPs from 507.19 G to 559.66 G, corresponding to increases of approximately 12.0% and 10.3%, respectively. The inference latency increases from 137.06 ms to 181.20 ms per image, while the peak inference memory increases from 0.88 GB to 1.29 GB. Meanwhile, the mIoU improves from 84.10% to 85.01%. The module-wise comparison shows that HFSM accounts for most of the additional computational cost because it involves fuzzy clustering, hypergraph construction, and node–hyperedge message propagation, whereas MCM introduces relatively limited additional overhead. These results indicate that the improved segmentation performance is accompanied by additional computational and memory costs, which should be considered in practical deployment.

**Limitations.** Several limitations remain in the current framework. First, although HyperNet consistently improves the baseline in the conducted ablation experiments, the numerical margins over some existing methods are relatively small, and the proposed method does not achieve the best result on every metric, as illustrated by the higher F1 score of SFA-Net on Potsdam. Second, the non-monotonic cluster-number analysis indicates that hypergraph construction is sensitive to the cluster configuration, suggesting that a more adaptive strategy for determining the hypergraph structure would be desirable. Third, HFSM introduces additional computational and memory overhead, which may limit its applicability in resource-constrained scenarios. Finally, the class-wise and failure-case analyses show that vegetation-related categories and small or fragmented objects remain challenging for the current model. Future work will therefore focus on lightweight and adaptive hypergraph construction, reducing sensitivity to clustering hyperparameters, and improving the representation of fine-grained and ambiguous semantic regions.

## 7. Conclusions

This paper proposes HyperNet, a hypergraph-driven framework for remote sensing semantic segmentation that models higher-order heterogeneous spatial relationships among geographically distributed regions. HFSM constructs adaptive hyperedges through fuzzy clustering to learn higher-order relational information, while MCM enhances robust multi-scale feature interaction. Experiments on the LoveDA, Vaihingen, and Potsdam datasets demonstrate state-of-the-art mIoU performance, with mIoU scores of 54.70%, 85.01%, and 87.64%, respectively. Ablation studies further confirm the individual and complementary contributions of HFSM and MCM.

Several limitations remain. The current experiments do not separately quantify specific topological or functional relationships. In addition, HFSM introduces additional computational overhead, and the constructed hypergraph depends on the clustering strategy and associated hyperparameters. The performance on very small and fragmented objects also requires further investigation. Future work will focus on lightweight and robust hypergraph modeling, object-scale-aware representation learning, and more direct relation-level analysis.

## Figures and Tables

**Table 1:** Geographical and imaging characteristics of the datasets used in this study. The reported coordinates represent approximate central locations of the corresponding study areas rather than the coordinates of individual image tiles.

Dataset	Study Area	Latitude	Longitude	GSD	Image Source/Product	Bands Used	Atmospheric Correction
Vaihingen	Vaihingen, Germany	48.936° N	8.960° E	0.09 m	Airborne TOP	NIR, R, G	Not specified
Potsdam	Potsdam, Germany	52.399° N	13.066° E	0.05 m	Airborne TOP	R, G, B	Not specified
LoveDA	Nanjing, China	32.062° N	118.778° E	0.30 m	Google Earth	R, G, B	Not specified
LoveDA	Changzhou, China	31.774° N	119.954° E	0.30 m	Google Earth	R, G, B	Not specified
LoveDA	Wuhan, China	30.583° N	114.267° E	0.30 m	Google Earth	R, G, B	Not specified

**Table 5:** Per-class IoU and F1 score comparison on the Vaihingen dataset. (Imp. Surf = Impervious Surfaces, Low Veg = Low Vegetation).

Method	Imp. Surf		Building		Low Veg		Tree		Car	
	IoU	F1	IoU	F1	IoU	F1	IoU	F1	IoU	F1
FT-UNetFormer	87.79	93.50	92.31	96.00	**75.00**	**85.60**	**83.15**	**90.80**	82.77	90.40
HyperNet (Ours)	**94.23**	**97.03**	**92.48**	**96.09**	73.19	84.52	81.94	90.07	**83.18**	**90.82**

*Note:* The best results are highlighted in bold.

**Table 6:** Effectiveness of different modules in HyperNet on the Vaihingen dataset. Results are reported as mean ± standard deviation over three independent runs.

Baseline	HFSM	MCM	F1 (%)	mIoU (%)	ΔmIoU (%)	OA (%)
✓			91.32±0.18	84.10±0.22	–	91.62±0.15
✓	✓		91.63±0.12	84.81±0.24	+0.71	93.47±0.10
✓		✓	91.52±0.20	84.62±0.16	+0.52	92.31±0.17
✓	✓	✓	91.70±0.18	85.01±0.11	**+0.91**	93.50±0.06

*Note:* ✓ indicates that the corresponding component is included. The best results are highlighted in bold.

**Table 7:** Ablation results of HFSM and MCM on the LoveDA dataset. Results are reported as mean ± standard deviation over three independent runs.

Baseline	HFSM	MCM	mIoU (%)	ΔmIoU (%)
✓			53.84±0.29	–
✓	✓		54.32±0.34	+0.48
✓		✓	54.18±0.16	+0.34
✓	✓	✓	54.70±0.12	**+0.86**

*Note:* ✓ indicates that the corresponding component is included. The best results are highlighted in bold.

**Table 9:** Sensitivity analysis of the loss weighting coefficient λ on the Vaihingen dataset.

λ	F1 (%)	mIoU (%)	OA (%)
0.5	91.48	84.67	93.29
1.0	91.60	84.86	93.41
1.5	**91.70**	**85.01**	**93.50**
2.0	91.66	84.93	93.46
2.5	91.53	84.74	93.31

*Note:* The best results are highlighted in bold.

**Table 10:** Computational complexity and segmentation performance of different configurations on the Vaihingen dataset.

Method	Params (M)	FLOPs (G)	Inference Latency (ms)	Peak Inference Memory (GB)	mIoU (%)
Baseline	95.98	507.19	137.06	0.88	84.10
Baseline+HFSM	107.42	558.73	177.84	1.24	84.81
Baseline+MCM	96.03	508.12	140.12	0.92	84.62
HyperNet	107.47	559.66	181.20	1.29	85.01

## Data Availability

The original contributions presented in this study are included in the article. Further inquiries can be directed to the corresponding author.

## References

[1] Liu P. (2025). A multimodal fusion framework for semantic segmentation of remote sensing based on multilevel feature fusion learning. Neurocomputing.

[2] Zeng W., Cheng M., Yuan Z., Dai W., Wu Y., Liu W., Wang C. (2024). Domain adaptive remote sensing image semantic segmentation with prototype guidance. Neurocomputing.

[3] Qin Z., Yu D., Luo C., Chen Z. Sliced Wasserstein Bridge for Open-Vocabulary Video Instance Segmentation. Proceedings of the IEEE/CVF International Conference on Computer Vision.

[4] Chu Y., Ye M., Qian Y. (2024). Fine-Grained Image Recognition Methods and Their Applications in Remote Sensing Images: A Review. IEEE J. Sel. Top. Appl. Earth Obs. Remote Sens..

[5] Li J., Cai Y., Li Q., Kou M., Zhang T. (2024). A review of remote sensing image segmentation by deep learning methods. Int. J. Digit. Earth.

[6] Alam M., Wang J.F., Guangpei C., Yunrong L., Chen Y. (2021). Convolutional Neural Network for the Semantic Segmentation of Remote Sensing Images. Mob. Netw. Appl..

[7] He X., Zhou Y., Zhao J., Zhang D., Yao R., Xue Y. (2022). Swin Transformer Embedding UNet for Remote Sensing Image Semantic Segmentation. IEEE Trans. Geosci. Remote Sens..

[8] Wang L., Li R., Zhang C., Fang S., Duan C., Meng X., Atkinson P.M. (2022). UNetFormer: A UNet-like transformer for efficient semantic segmentation of remote sensing urban scene imagery. ISPRS J. Photogramm. Remote Sens..

[9] Cai D., Song M., Sun C., Zhang B., Hong S., Li H. Hypergraph Structure Learning for Hypergraph Neural Networks. Proceedings of the IJCAI.

[10] Ouyang S., Li Y. (2020). Combining Deep Semantic Segmentation Network and Graph Convolutional Neural Network for Semantic Segmentation of Remote Sensing Imagery. Remote Sens..

[11] Qin Z., Yu D., Shi Y., Wang Q., Chen Z. Video Instance Segmentation by Weighted Structure Inference. Proceedings of the 33rd ACM International Conference on Multimedia.

[12] Shelhamer E., Long J., Darrell T. (2017). Fully Convolutional Networks for Semantic Segmentation. IEEE Trans. Pattern Anal. Mach. Intell..

[13] Ronneberger O., Fischer P., Brox T. (2015). U-net: Convolutional networks for biomedical image segmentation. Medical Image Computing and Computer-Assisted Intervention–MICCAI 2015.

[14] Yang Z., Zhou D., Yang Y., Zhang J., Chen Z. (2023). Road extraction from satellite imagery by road context and full-stage feature. IEEE Geosci. Remote Sens. Lett..

[15] Liu M., Dan J., Lu Z., Yu Y., Li Y., Li X. (2024). CM-UNet: Hybrid CNN-Mamba UNet for Remote Sensing Image Semantic Segmentation. arXiv.

[16] John D., Zhang C. (2022). An attention-based U-Net for detecting deforestation within satellite sensor imagery. Int. J. Appl. Earth Obs. Geoinf..

[17] Fu J., Liu J., Tian H., Li Y., Bao Y., Fang Z., Lu H. Dual attention network for scene segmentation. Proceedings of the IEEE/CVF Conference on Computer Vision and Pattern Recognition.

[18] Yu C., Gao C., Wang J., Yu G., Shen C., Sang N. (2021). BiSeNet V2: Bilateral Network with Guided Aggregation for Real-Time Semantic Segmentation. Int. J. Comput. Vis..

[19] Wang Y., Yang L., Liu X., Yan P. (2024). An improved semantic segmentation algorithm for high-resolution remote sensing images based on DeepLabv3+. Sci. Rep..

[20] Ma X., Lian R., Wu Z., Guo H., Yang F., Ma M., Wu S., Du Z., Zhang W., Song S. (2025). LOGCAN++: Adaptive Local-Global Class-Aware Network for Semantic Segmentation of Remote Sensing Images. IEEE Trans. Geosci. Remote Sens..

[21] Han K., Wang Y., Chen H., Chen X., Guo J., Liu Z., Tang Y., Xiao A., Xu C., Xu Y. (2023). A Survey on Vision Transformer. IEEE Trans. Pattern Anal. Mach. Intell..

[22] Wang W., Xie E., Li X., Fan D.P., Song K., Liang D., Lu T., Luo P., Shao L. Pyramid vision transformer: A versatile backbone for dense prediction without convolutions. Proceedings of the IEEE/CVF International Conference on Computer Vision.

[23] Liu Z., Lin Y., Cao Y., Hu H., Wei Y., Zhang Z., Lin S., Guo B. Swin Transformer: Hierarchical Vision Transformer Using Shifted Windows. Proceedings of the IEEE/CVF International Conference on Computer Vision (ICCV).

[24] Carion N., Massa F., Synnaeve G., Usunier N., Kirillov A., Zagoruyko S. End-to-end object detection with transformers. Proceedings of the European Conference on Computer Vision.

[25] Strudel R., Garcia R., Laptev I., Schmid C. Segmenter: Transformer for Semantic Segmentation. Proceedings of the IEEE/CVF International Conference on Computer Vision (ICCV).

[26] Li H., Li L., Zhao L., Liu F. (2024). ResU-Former: Advancing Remote Sensing Image Segmentation with Swin Residual Transformer for Precise Global–Local Feature Recognition and Visual–Semantic Space Learning. Electronics.

[27] Yuan Y., Fu R., Huang L., Lin W., Zhang C., Chen X., Wang J. (2021). HRFormer: High-Resolution Transformer for Dense Prediction. Advances in Neural Information Processing Systems 34 (NeurIPS 2021).

[28] Fan L., Zhou Y., Liu H., Li Y., Cao D. (2023). Combining Swin Transformer with UNet for Remote Sensing Image Semantic Segmentation. IEEE Trans. Geosci. Remote Sens..

[29] Cui W., Yao M., Hao Y., Wang Z., He X., Wu W., Li J., Zhao H., Xia C., Wang J. (2021). Knowledge and geo-object based graph convolutional network for remote sensing semantic segmentation. Sensors.

[30] Kang X., Hong Y., Duan P., Li S. (2024). Fusion of hierarchical class graphs for remote sensing semantic segmentation. Inf. Fusion.

[31] Zhao Y., Qiu L., Yang Z., Chen Y., Zhang Y. (2025). MGF-GCN: Multimodal interaction Mamba-aided graph convolutional fusion network for semantic segmentation of remote sensing images. Inf. Fusion.

[32] Wu J., Fu R., Liu Q., Ni W., Cheng K., Li B., Sun Y. (2023). A dual neighborhood hypergraph neural network for change detection in VHR remote sensing images. Remote Sens..

[33] Jing W., Zhang W., Di D., Li C., Emam M., Mian A. (2025). Hypergraph biformer for semantic segmentation of high-resolution remote sensing images. IEEE Trans. Geosci. Remote Sens..

[34] Wang J., Zheng Z., Ma A., Lu X., Zhong Y. (2021). LoveDA: A Remote Sensing Land-Cover Dataset for Domain Adaptive Semantic Segmentation. Neural Information Processing Systems Track on Datasets and Benchmarks.

[35] Li Y., Li X., Dai Y., Hou Q., Liu L., Liu Y., Cheng M.M., Yang J. (2024). LSKNet: A Foundation Lightweight Backbone for Remote Sensing. Int. J. Comput. Vis..

[36] Chen Y., Fang P., Zhong X., Yu J., Zhang X., Li T. (2024). Hi-ResNet: Edge Detail Enhancement for High-Resolution Remote Sensing Segmentation. IEEE J. Sel. Top. Appl. Earth Obs. Remote Sens..

[37] Lu W., Chen S.B., Ding C.H.Q., Tang J., Luo B. (2025). LWGANet: A Lightweight Group Attention Backbone for Remote Sensing Visual Tasks. arXiv.

[38] Wang D., Zhang Q., Xu Y., Zhang J., Du B., Tao D., Zhang L. (2023). Advancing Plain Vision Transformer Toward Remote Sensing Foundation Model. IEEE Trans. Geosci. Remote Sens..

[39] Cha K., Seo J., Lee T. (2024). A Billion-scale Foundation Model for Remote Sensing Images. IEEE J. Sel. Top. Appl. Earth Obs. Remote Sens..

[40] Hanyu T., Yamazaki K., Tran M., McCann R.A., Liao H., Rainwater C., Adkins M., Cothren J., Le N. (2024). AerialFormer: Multi-Resolution Transformer for Aerial Image Segmentation. Remote Sens..

[41] Wang F., Wang H., Wang D., Guo Z., Zhong Z., Lan L., Yang W., Zhang J. (2024). Harnessing Massive Satellite Imagery with Efficient Masked Image Modeling. arXiv.

[42] Wang D., Zhang J., Xu M., Liu L., Wang D., Gao E., Han C., Guo H., Du B., Tao D. (2024). MTP: Advancing remote sensing foundation model via multitask pretraining. IEEE J. Sel. Top. Appl. Earth Obs. Remote Sens..

[43] Lu W., Chen S.B., Shu Q.L., Tang J., Luo B. (2024). DecoupleNet: A Lightweight Backbone Network with Efficient Feature Decoupling for Remote Sensing Visual Tasks. IEEE Trans. Geosci. Remote Sens..

[44] Li R., Zheng S., Zhang C., Duan C., Wang L., Atkinson P.M. (2021). ABCNet: Attentive bilateral contextual network for efficient semantic segmentation of Fine-Resolution remotely sensed imagery. ISPRS J. Photogramm. Remote Sens..

[45] Li R., Zheng S., Zhang C., Duan C., Su J., Wang L., Atkinson P.M. (2021). Multiattention network for semantic segmentation of fine-resolution remote sensing images. IEEE Trans. Geosci. Remote Sens..

[46] Wu H., Huang P., Zhang M., Tang W., Yu X. (2023). CMTFNet: CNN and Multiscale Transformer Fusion Network for Remote-Sensing Image Semantic Segmentation. IEEE Trans. Geosci. Remote Sens..

[47] Kim B.J., Kim S.W. (2024). Stochastic Subsampling with Average Pooling. arXiv.

[48] Zhang X., Weng Z., Zhu P., Han X., Zhu J., Jiao L. (2024). ESDINet: Efficient Shallow-Deep Interaction Network for Semantic Segmentation of High-Resolution Aerial Images. IEEE Trans. Geosci. Remote Sens..

[49] Li B., Zhang Y., Zhang Y., Li B., Li Z. (2024). Dual-Path Feature Fusion Network for Semantic Segmentation of Remote Sensing Images. IEEE Geosci. Remote Sens. Lett..

[50] Ma X., Zhang X., Pun M.O. (2024). RS3Mamba: Visual State Space Model for Remote Sensing Image Semantic Segmentation. IEEE Geosci. Remote Sens. Lett..

[51] Wang L., Li R., Wang D., Duan C., Wang T., Meng X. (2021). Transformer meets convolution: A bilateral awareness network for semantic segmentation of very fine resolution urban scene images. Remote Sens..

[52] Wang L., Li R., Duan C., Zhang C., Meng X., Fang S. (2022). A novel transformer based semantic segmentation scheme for fine-resolution remote sensing images. IEEE Geosci. Remote Sens. Lett..

[53] Hwang G., Jeong J., Lee S.J. (2024). SFA-Net: Semantic feature adjustment network for remote sensing image segmentation. Remote Sens..

[54] Li R., Zheng S., Duan C., Su J., Zhang C. (2022). Multistage Attention ResU-Net for Semantic Segmentation of Fine-Resolution Remote Sensing Images. IEEE Geosci. Remote Sens. Lett..

[55] Cheng B., Misra I., Schwing A.G., Kirillov A., Girdhar R. Masked-attention mask transformer for universal image segmentation. Proceedings of the IEEE/CVF Conference on Computer Vision and Pattern Recognition.

[56] Wang Q., Luo X., Feng J., Zhang G., Jia X., Yin J. (2023). Multiscale Prototype Contrast Network for High-Resolution Aerial Imagery Semantic Segmentation. IEEE Trans. Geosci. Remote Sens..

[57] Zhao S., Chen H., Zhang X., Xiao P., Bai L., Ouyang W. (2024). RS-Mamba for Large Remote Sensing Image Dense Prediction. IEEE Trans. Geosci. Remote Sens..

[58] Wang L., Li D., Dong S., Meng X., Zhang X., Hong D. (2024). PyramidMamba: Rethinking pyramid feature fusion with selective space state model for semantic segmentation of remote sensing imagery. arXiv.

[59] Zhu Q., Cai Y., Fang Y., Yang Y., Chen C., Fan L., Nguyen A. (2024). Samba: Semantic segmentation of remotely sensed images with state space model. Heliyon.

[60] Wang J., Li J., Fan G., Ju Y., Fang X., Kot A.C. (2025). Prototype-Driven Structure Synergy Network for Remote Sensing Images Segmentation. IEEE Trans. Geosci. Remote Sens..

